# A biologically constrained agent-based model of cancer stem cell dynamics with reinforcement learning-guided adaptive radiotherapy

**DOI:** 10.1371/journal.pone.0340426

**Published:** 2026-02-05

**Authors:** Mina Lagzian, S. Ehsan Razavi, Reyhane Kardehi Moghaddam

**Affiliations:** Department of Electrical Engineering, Ma.C., Islamic Azad University, Mashhad, Iran; University of South Carolina, UNITED STATES OF AMERICA

## Abstract

Cancer stem cells (CSCs) represent a rare but critical subpopulation within tumors, driving recurrence, resistance to therapy, and aggressive growth. To better understand CSC behavior in solid tumors, we developed a biologically constrained agent-based model (ABM) that simulates tumor progression initiated from a single CSC. The model incorporates essential microenvironmental factors—including oxygen diffusion, spatial limitations, stochastic migration, and cell cycle dynamics—allowing for high-resolution simulation of tumor development and intra tumoral heterogeneity. While this work does not aim to fully optimize therapy for clinical application, it provides a flexible, scalable simulation environment where adaptive treatment strategies can be tested. To extend a biological model toward intelligent treatment, we integrated a reinforcement learning (Q-learning) component that adaptively adjusts radiation dosage based on real-time CSC localization and microenvironmental feedback. This component is currently presented as a proof-of-concept to demonstrate feasibility, and its optimization and convergence analysis will be explored in future studies. Our results suggest that reinforcement learning, when integrated with a biologically grounded ABM, can guide adaptive and more personalized radiotherapy strategies.

## Introduction

Cancer is a complex, heterogeneous disease, characterized by uncontrolled cell proliferation, genetic transformation, and drug resistance. Cancer stem cells (CSCs)- a subpopulation with self-renewal potential and the ability to cause recurrence of cancer even after aggressive therapy [1–3]. CSCs are resistant to conventional cancer therapies such as chemotherapy and radiotherapy [4,5].

To understand and counteract CSC-driven tumor behavior, **in silico models** have become increasingly important. Among these, **agent-based models (ABMs)** are particularly powerful due to their ability to simulate individual cell behavior, stochastic processes, and environment interactions over time [6,7]. Previous agent-based models (ABMs) have captured tumor heterogeneity and spatial dynamics under microenvironmental constraints such as hypoxia and nutrient gradients [8,9]. These models have since been extended to include microenvironmental factors like hypoxia and nutrient gradients [10].

Previous works by Lagzian and colleagues used agent-based frameworks to identify key behavioral parameters affecting tumor expansion [11–14]. Unlike Jalalimanesh et al. [15], who optimized dose schedules in a mathematical framework, our study embeds RL within a biologically detailed, CSC-coupled ABM so that the agent’s state, actions, and rewards are directly informed by tumor biology.

Recent studies, such as Wang et al. (2024), explored RL for adaptive cancer therapy, but without CSC-level biological coupling. Our work advances this direction by embedding RL into a biologically grounded ABM that connects AI-driven decision-making with realistic cellular constraints [16,17]. For example, Wang et al. (2024) implemented an RL-guided immunotherapy framework, highlighting the potential of intelligent decision-making in oncology but without detailed CSC-level biological coupling. Our work builds on these advances by embedding RL into a biologically detailed ABM, thereby linking AI-driven adaptation with realistic cellular constraints.

The primary novelty of this work lies in the integration of a **biologically constrained agent-based model (ABM)**explicitly incorporating oxygen diffusion, spatial competition, and the hierarchical CSC/non-CSC dynamics—with a **reinforcement learning (Q-learning)** framework for adaptive radiotherapy. Unlike previous studies that applied RL mainly to mathematical dose-optimization problems without biological feedback, our model links the learning process directly to the tumor’s biological state.

Here, the *state* vector of the agent is defined by biologically meaningful quantities, including the spatial distribution of CSCs across four quadrants, mean oxygen concentration, and overall tumor area, while the *action* space combines discrete dose levels with directional irradiation gates.

Furthermore, the reward function explicitly balances CSC eradication and preservation of healthy tissue. A quantitative comparison with a constant-dose radiotherapy protocol is also provided to demonstrate the adaptive advantage of the proposed approach.

In this study, we provide a computational basis that prioritizes biological realism and algorithmic feasibility, rather than aiming for a fully optimized clinical implementation.

To our knowledge, this is among the first modeling frameworks to integrate reinforcement learning within a CSC-focused ABM, offering a conceptual bridge between biological modeling and intelligent therapy optimization. While this paper focuses primarily on validating the simulation framework and assessing its biological plausibility, a more detailed study of the learning algorithm’s behavior and therapeutic outcomes will be presented in future work. To validate the model, we compare the simulated tumor area dynamics with **experimental CSC data extracted from Norton et al. [10]**, demonstrating the biological plausibility and computational strength of our framework. This study serves as a **2D proof-of-concept** designed to balance biological realism and computational tractability. Several simplifications were intentionally adopted: (a) immune interactions and detailed molecular signaling pathways were not explicitly modeled;

(b) oxygen diffusion and radiosensitivity parameters were selected within experimentally validated biological ranges; and (c) unlimited CSC proliferation was assumed to isolate intrinsic stem-cell dynamics without confounding effects. These simplifications intentionally narrow the scope of this study to comparative control strategies and emergent-behavior analysis.

Although the present framework is validated primarily against preclinical in vivo data, its principles are translationally relevant. Mapping the simulated dose fractions to clinical radiotherapy schedules will require patient-specific calibration and consideration of tissue toxicity and dose- volume constraints. Nevertheless, the concept of **feedback-driven, CSC-targeted adaptive dosing** offers a promising direction for the development of personalized treatment planning systems. In future work, the model will be extended to 3D geometries and coupled with patient-derived microenvironmental data to enhance its clinical interpretability.

Based on these motivations, the goal of this study is to develop a biologically constrained agent-based model (ABM) integrated with reinforcement learning (Q-learning) for adaptive radiotherapy. Specifically, the model aims to (1) simulate CSC and non-CSC dynamics under oxygen and spatial constraints, and (2) enable adaptive radiation dosing based on real-time tumor feedback. We hypothesize that RL-guided radiotherapy achieves better CSC suppression and lower total dose compared to conventional constant-dose protocols.

## Method and materials

### Simulation domain & initialization

In this study, we proposed an in-silico simulation of the cancerous tumor growth process using a constrained agent-based model (ABM). The simulation was implemented in Python to model the behavior of cancer stem cells within a virtual cellular tissue environment. Below, we outline the key steps and components of the modeling process:

1. We simulate a 2D tissue lattice (350 × 350 sites) with unit lattice spacing (Δx = 1, arbitrary units) and apply no-flux boundary conditions to the diffusion fields. Unless stated otherwise, results were confirmed on a smaller **100 × 100** grid for calibration and run-time profiling. A single CSC is placed at a random lattice site at **t = 0** as the initial progenitor. We fix the random seed to **SEED = 42** to ensure reproducibility. Time advances in discrete steps of Δt = 1 hour, and the total simulated period is 480 hours (=20 days), unless stated otherwise.
2. Oxygen availability within the simulated tissue is governed by a network of randomly distributed microchannels that mimic small blood vessels. Their centers are generated following a Gaussian spatial distribution, and both the number and density of channels are adjustable parameters. Oxygen concentration O(𝐱,t)changes over time according to a standard reaction–diffusion equation:

$$\frac{\partial O}{\partial t}=D\nabla^{\text{2}}O-k\;C(𝐱,t)+S(𝐱)$$

where D is the diffusion coefficient, krepresents cellular oxygen consumption, and S(𝐱)denotes local oxygen sources at microchannel sites. The equation is numerically solved using an explicit five-point stencil under no-flux boundary conditions. Oxygen diffusion and consumption were modeled using a simplified reaction-diffusion equation and adapted from [10,18,19]. The updated oxygen map is then coupled to the agent-based model at every time step to influence cell proliferation, death, and radiosensitivity. The diffusion and consumption parameters used in the oxygen model were carefully selected to ensure both numerical stability and biological realism. Table 1 summarizes the key constants and their ranges:

**Table 1:** Oxygen diffusion and consumption parameters.

Symbol	Parameter	Meaning/ Biological Role	Value/ Range	Unit	Reference/ Note
**D**	Diffusion coefficient	Controls rate of oxygen spread through tissue	0.05–0.20	a.u. · site² / h	Within experimentally observed ranges (Grimes et al. 2016)
**k**	Cellular consumption rate	Oxygen uptake per cell per unit time	0.001–0.01	a.u. / cell · h	Higher for proliferating cells
**S**	Source intensity of microchannel	Oxygen supplied at vessel locations	0.2–1.0	a.u. / h	Applied to Gaussian-distributed microchannels
$O_{max}$	Maximum oxygen concentration	Saturation level near microchannels	1.0	a.u.	Normalized reference concentration
$O_{min}$	Minimum viable oxygen	Threshold for proliferation shutdown	0.05–0.1	a.u.	Below this, cell division ceases
$O_{crit}$	Hypoxia threshold	Level below which cell death probability increases	0.02–0.05	a.u.	Triggers hypoxia-induced death
Δx	Lattice spacing	Spatial resolution of diffusion grid	1	a.u.	Matches ABM lattice spacing
Δt	Time step	Numerical integration interval	1	h	Satisfies ($D\frac{\Delta t}{{\Delta x}^{\text{2}}}$≤ 0.25) (stability)

Parameter ranges were selected to satisfy numerical stability and biological plausibility according to Grimes et al. (2016) and Norton et al. (2014).

1. Cell Initialization: A single cancer stem cell was randomly placed within the grid as the progenitor cell that initiates tumor growth.
2. Cell Age Definition: Each cell was assigned an age parameter based on its cell cycle phase.
3. Simulation Execution: The growth process of cancer stem cells was simulated over time, tracking cell proliferation, migration, and death.
4. Tumor Area Calculation: We computed the cross-sectional area occupied by cancerous cells to assess tumor expansion and inform radiotherapy strategies.

**Fig 1:**
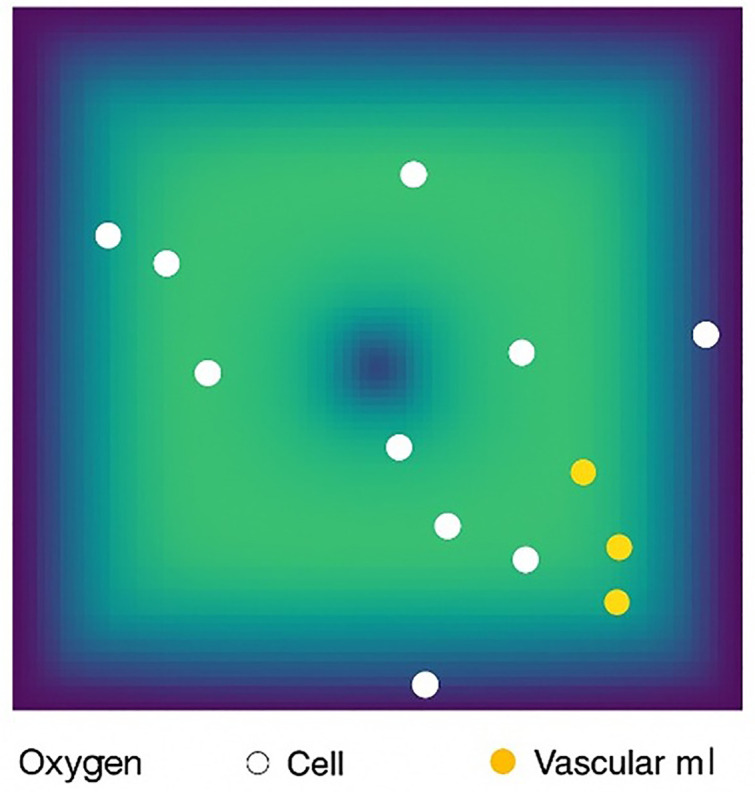
Schematic of the simulation environment including microchannel distribution. A 2D tissued grid with randomly positioned cancer stem cell (CSC) and oxygen microchannels shown as white dots. The model simulates CSC behavior under spatial and oxygen constraints.

As shown in Fig 1, the simulated tissue environment includes:

This is a simulated tissue in which we randomly placed a cancer stem cell and simulated how it grows.

### Constrained agent-based model

1-We selected our agent-based modeling for its ability to capture the stochastic and individual behaviors of cancer cells. This constrained ABM enhances accuracy by incorporating physiological limitations observed in vivo. Each cancer cell was modeled as an agent with attributes including:

a. - Type (stem cell, (C), or non-stem cell, (P)),
b. - Position within the grid,
c. - Lifespan,
d. - Behaviors (migration, division, death).

### Model constraints

To capture realistic vivo tumor behavior, ten biologically grounded constraints were defined within the agent-based model. These include rules for symmetric and asymmetric division of cancer stem cells (Constraints 1 and 5), proliferation of non-stem cells (Constraint 6), spatial and resource competition among neighboring cells (Constraints 2 and 7), oxygen- and nutrient-limited growth (Constraints 3 and 8), and natural cell death or phenotype switching (Constraints 4, 9, and 10). Together, these constraints ensure that cell division, migration, and death occur under physiologically plausible conditions and maintain the hierarchical balance between CSCs and non-CSCs.

### Key parameters

#### Migration probability.

Derived from references [20–22], this parameter dictates the likelihood of cell movement to one of eight neighboring positions. - Symmetric Division Probability: This determines the proportion of cancer stem cells in the tumor, a critical factor in growth dynamics. - Oxygen Concentration and Cell Cycle Phase: These were imposed as environmental constraints, influencing cell behavior.

#### Proliferation rate.

CSC proliferation was modeled without an intrinsic replicative limit to isolate stem-cell–driven expansion; environmental limits (oxygen, space) still curb growth. reflecting their high regenerative potential. The unlimited proliferation assumption was adopted to focus on the intrinsic growth dynamics of CSCs under idealized conditions, enabling clearer interpretation of their contribution to tumor expansion, while also reducing computational complexity for the continuation and broader objectives of this research.

### Simulation details

We implemented a stochastic simulation framework, incorporating statistical distributions—such as Gaussian patterns for oxygen diffusion and cell migration—to better reflect biological variability. Building on insights from our earlier study [11], we specifically examined two influential parameters identified through the Taguchi method: migration probability and symmetric division rate. These were selected for closer analysis due to their strong impact on tumor dynamics.

### Sensitivity analysis

#### To evaluate the influence of key biological parameters on tumor development and cancer stem cell dynamics, a sensitivity analysis was conducted.

This analysis aimed to determine how variations in specific model inputs affect critical tumor characteristics, including CSC population size, tumor spatial expansion, and cellular migration behavior. In particular, we focused on three biologically meaningful parameters: the symmetric division probability of CSCs (a00), the asymmetric division probability leading to non-stem progeny (a01), and the migration probability of individual cells. Each parameter was systematically varied across a biologically plausible range, while other factors were held constant. By examining resulting changes in CSC count, tumor area (measured via convex hull), and average migration frequency, we were able to quantify the role of each parameter in driving tumor aggressiveness and spatial heterogeneity. Table 2, shows the sensitivity analysis of a00 with 100 stage simulation:

**Table 2:** Sensitivity analysis of a00.

Amount of a00	Final number of alive CSCs	Final area of tumor
0.05	11	7.0
0.10	37	29.0
0.15	41	32.0
0.20	42	32.5
0.25	47	35.0

When the value of a00 increases, the number of alive cancer stem cells and the overall tumor size steadily rise. Beyond a00 = 0.15, the growth in cell numbers becomes slower but remains stable. It may indicate a state of relative saturation or spatial competition. This analysis remarks that symmetric division of CSCs has a direct impact on the stability and expansion of tumor progression.

Below, the sensitivity analysis of two other parameter that we considered in our previous work [11], is brought: **Asymmetric division of CSCs into one CSC and one non-stem cell, controlled by parameter a01, showed a suppressive effect on CSC population while increasing tumor heterogeneity.** Increasing a01 led to a decline in the number of CSCs—from 14 to 4 cells—as more divisions favored non-stem lineages. The tumor area also decreased from 23.0 to 10.5 units across this range, highlighting that reduced CSC self-renewal limits spatial growth. This confirms that asymmetric division promotes diversification of cell types at the cost of stemness maintenance, in line with the CSC hierarchy hypothesis. The results are brought in Table 3.

**Table 3:** Sensitivity analysis of a01.

Final tumor area	Final number of non-CSC	Final number of CSC	Amount of a01
**23.0**	18	14	0.20
**15.0**	16	6	0.30
**10.5**	13	4	0.40
**11.0**	13	5	0.50

At the end, we consider probability of migration parameter: As shown in Table 4, **the migration probability exhibited the most pronounced influence on tumor area expansion and overall cellular dynamics.** As migration increased from 0.0 to 0.4, the tumor area grew markedly from 40.5 to 568.0 units, while CSCs increased from 18 to 83 cells. Moreover, the average migration per cell rose steadily, confirming that enhanced motility enables spatial dispersion, cellular escape from overcrowded zones, and proliferation in new regions. These results highlight the importance of incorporating migration behavior into tumor models to capture metastatic potential and spatial heterogeneity.

**Table 4:** Sensitivity analysis of migration.

Mean of migration	Final tumor area	Final number of non-CSC	Final number of CSC	Prob. Of migration
0.000	40.5	41	18	0
0.518	179.0	135	35	0.1
0.988	270.0	203	44	0.2
1.559	431.5	251	62	0.3
2.266	568.0	361	83	0.4

**In summary, the sensitivity analysis confirmed the critical role of symmetric division, asymmetric differentiation, and cellular migration in shaping the tumor’s structure and dynamics.** Symmetric division (a00) emerged as a key driver of CSC population expansion, whereas asymmetric division (a01) limited stemness in favor of tumor heterogeneity. Meanwhile, increased migration probability led to extensive tumor spread and higher spatial complexity. These findings reinforce the biological plausibility of the model and suggest that subtle variations in cellular behaviors can significantly influence tumor progression. Thus, integrating such parameters into therapeutic planning or adaptive radiotherapy algorithms may improve treatment targeting and overall efficacy.

## Results

In this simulation, we proposed a constrained agent-based model to study the growth process of tumors and cancer stem cells. We considered the impact of two key factors influencing tumor development, identified using the Taguchi method: the **probability of migration** and the **symmetric division rate** of cancer stem cells. The simulation was implemented in Python, incorporating two biological constraints: **Tissue oxygen concentration** and the **cell cycle phase** of cancer cells.

**Fig 2:**
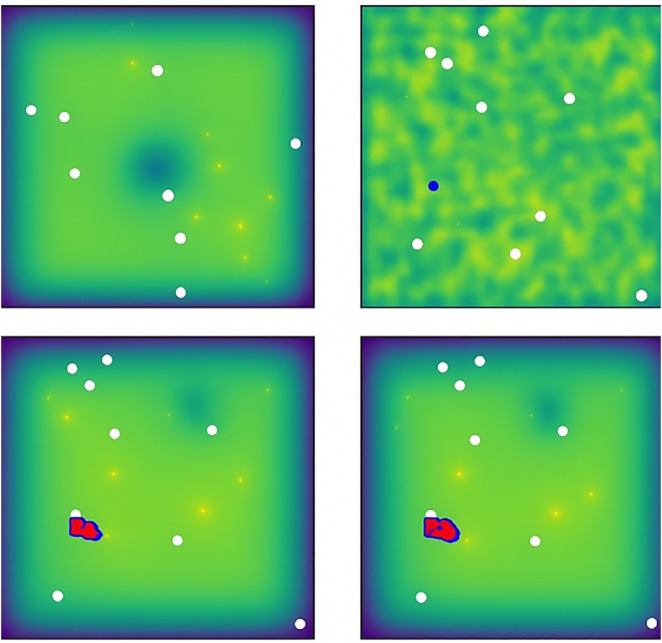
Simulated tumor growth initiated by a single cancer stem cell after 20 days. Blue represents CSCs; red indicates two phenotypic types of non-stem cells. The tumor exhibits hierarchical expansion and local heterogeneity.

Single cancer stem cell was initially placed as a progenitor in the simulation tissue. The tumor growth and formation process driven by this cancer stem cell was observed for a period of 20 days (20 days = 48 hours). As shown in Fig 2, the tumor grows hierarchically from the initial CSC:

We present the results of our simulation in the following section. We analyze the dynamics and variations of the following features over time:

- The number of cancer stem cells and non-stem cells
- The tumor-occupied area
- The cell migration patterns
- The oxygen concentration within the tissue

### The number of cancer stem and non-stem cells and total

**Fig 3:**
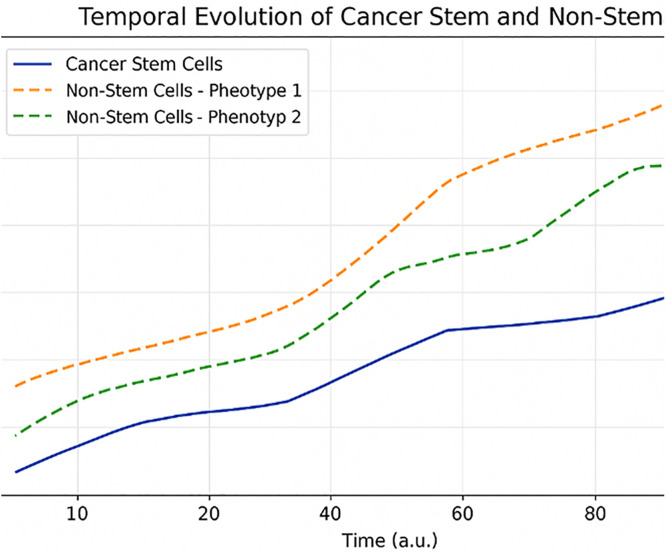
Time course of CSC and non-CSC population expansion. The number of CSCs and non-stem cancer cells over simulation time, reflecting asymmetric and symmetric division dynamics with emergence of hierarchical tumor structure.

Fig 3 shows the temporal dynamics of cancer stem cells (CSCs) and non-stem cancer cells generated by our agent-based model. The simulation shows a continuous increasing trend in both cell populations over time, such that after the initial phase, non-stem cells dominate stem cells in number. This pattern is consistent with the hierarchical structure of tumor growth, in which CSCs, despite their small numbers, play a central role in the initiation and perpetuation of the cancer process, while non-stem cells constitute the bulk of the tumor mass.

Interestingly, the observed nearly linear relationship between CSCs and non-stem cells arises from both symmetric and asymmetric division mechanisms. Asymmetric division, in particular, results in the production of one stem cell and one non-stem cell, which helps maintain the CSC population while at the same time promoting the gradual expansion of the non-stem fraction. This dynamic is well represented in the framework of an agent-based model, where each cell acts independently and under a set of probabilistic rules for division, death, and sometimes phenotype change. Importantly, these behaviors occur without imposing any predefined macroscopic structure on the model, demonstrating the power of agent-based approaches in simulating emergent tumor behaviors.

Local decreases in cell number are observed across pathways and are attributed to cell death and phenotype transition (cell switching). These events, which are inherently modeled in the ABM structure, introduce biologically realistic heterogeneity and nonlinearity into the population dynamics. In the case of non-stem cell subgroups (phenotype 1 and 2), we also observed a temporary decrease in their number due to phenotype-specific probabilities of death (i = 1,2), thus highlighting the capability of the model in accommodating enhanced intratumor heterogeneity.

In addition, the modularity of the agent-based model provides a means to potentially add future extra biological details, for instance, immune interactions, spatial heterogeneity, or cellular stress due to treatment. The dynamics observed are also consistent with known biological evidence of CSC-induced tumorigenesis, further validating the model structure and assumptions.

Taken altogether, these results underscore the predominant role of CSCs in regulating the global dynamics of the tumor. Despite their rarity, CSCs play a disproportionate role in tumor maintenance and progression. These results, therefore, have important implications for the engineering of targeted therapeutic strategies against cancer stem cells and highlight the effectiveness of agent-based modeling as a useful platform for investigating complex tumor dynamics.

### Cancer cells migration

The analysis of cancer cell migration in this study provides valuable insights into the dynamics of tumor progression. Based on the simulation results and corresponding diagrams, several important observations can be made:

1- **Non- uniform distribution of migration events**
2- **Distinct Behavior of Cancer Stem Cells (CSCs)**
3- **Localized Migration Pattern**
4- **Implications for Cancer Treatment**

**Fig 4:**
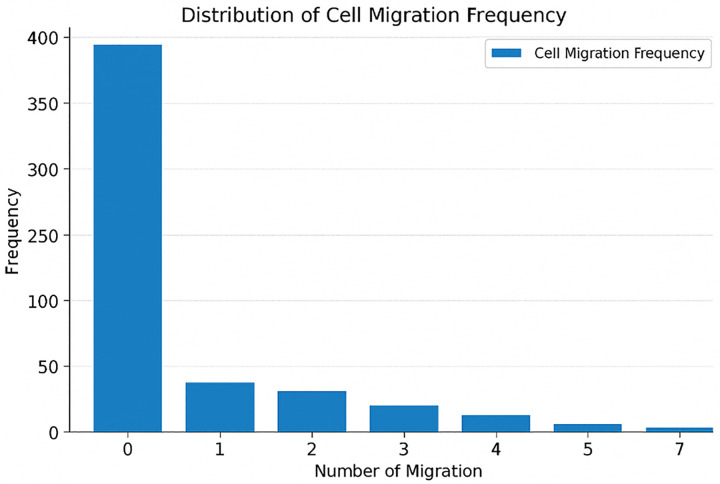
Histogram of cell migration frequency during tumor development. Most cells exhibit minimal migration, with a few undergoing multiple migrations- matching a power law distribution pattern.


As shown in Fig 4, most cells did not migrate during the simulation, and only a few cells migrated multiple times. This pattern is consistent with a power-law distribution, where the frequency of an event decreases rapidly as the number of events increases. This distribution is defined as follows and has a diagram like the one below.

$$f(x)=ax^{-k}$$

**Fig 5:**
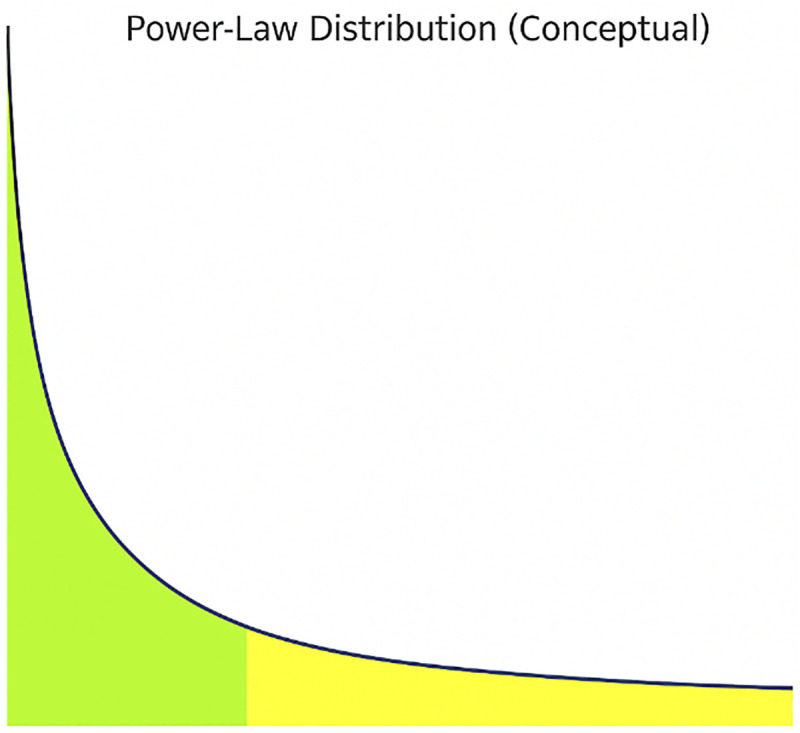
Reference power-law distribution for comparison. A generic power-law function is used as a conceptual benchmark for interpreting cell migration distribution in the simulation.

The parameters a and k are two distribution parameters and are estimable. As shown in Fig 5, a reference power-low distribution is used as a conceptual benchmark for interpreting the migration frequency patterns observed in our simulation.

Fig 5 represents a conceptual power-law distribution. In biological systems, similar distributions are common, such as in gene expression, protein networks, or cancer cell migration. The similarity between Figs 4 and 5 confirms the power-law behavior in migration frequency.

**Fig 6:**
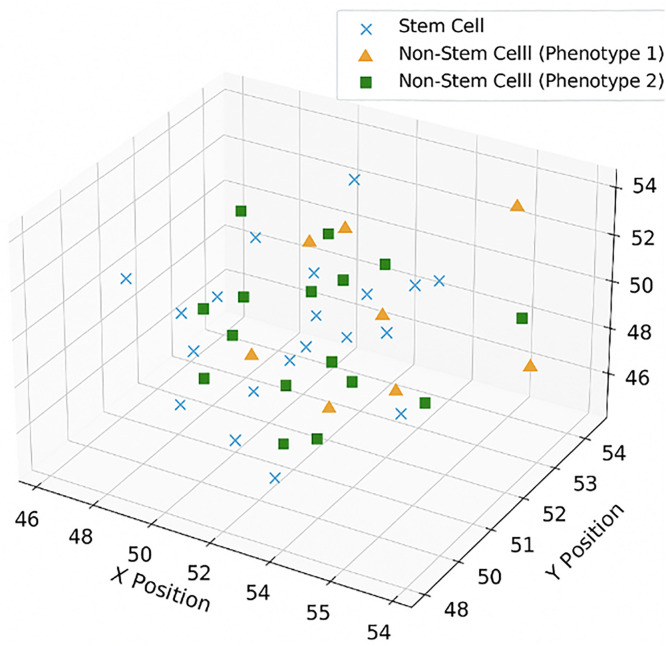
Spatial distribution of cell location by migration frequency. Cells with higher migration counts tend to spread further from the origin. CSCs are shown as circles, non-CSCs as triangles.

The above figure (Fig 6) illustrates the spatial distribution of cells “based on their migration frequency. Cancer stem cells (circles) show a higher average number of migrations compared to non-stem cancer cells (triangles). This is likely due to their early presence in the tissue, allowing more space and time for migration. Moreover, most cells remain in the neighborhood of the progenitor cell, suggesting a local spread pattern”.

### General analysis

In the simulation, cancer cell migration follows a power-law pattern: most cells move very little or don’t move at all. only a small number of cells migrate multiple times. This pattern is consistent with biological systems, where frequent long-distance migration is rare but can have a major impact on tumor evolution. Cancer stem cells (CSCs) tend to migrate more on average than non-stem cancer cells, probably because they appear earlier and have more space around them to move into. Additionally, migration usually happens close to the original (progenitor) cell rather than over long distances. These results show that CSCs might be especially important in tumor recurrence, because their movement beyond the main tumor site could help cancer come back. These notes prove that understanding and targeting cell mobility is crucial for effective treatments.

### Oxygen concentration in cross-section

**Fig 7:**
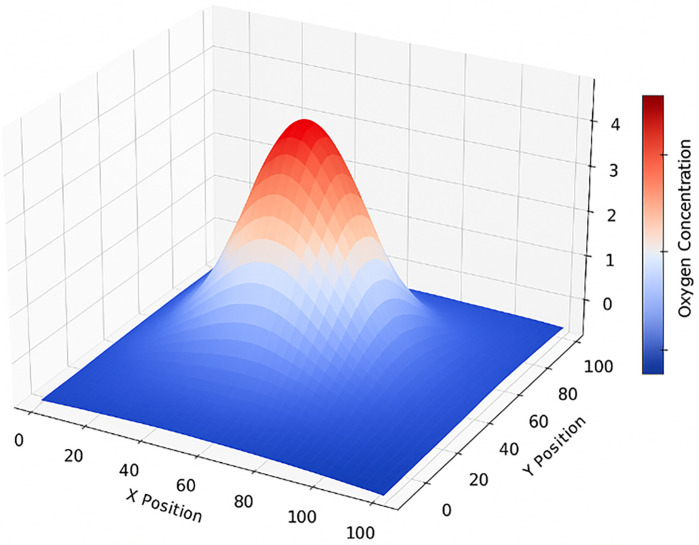
Cross-sectional oxygen concentration map in simulated tissue. Oxygen gradients reflect diffusion from microchannels and local consumption. Hypoxic zones emerge where tumor density increases.

**Fig 8:**
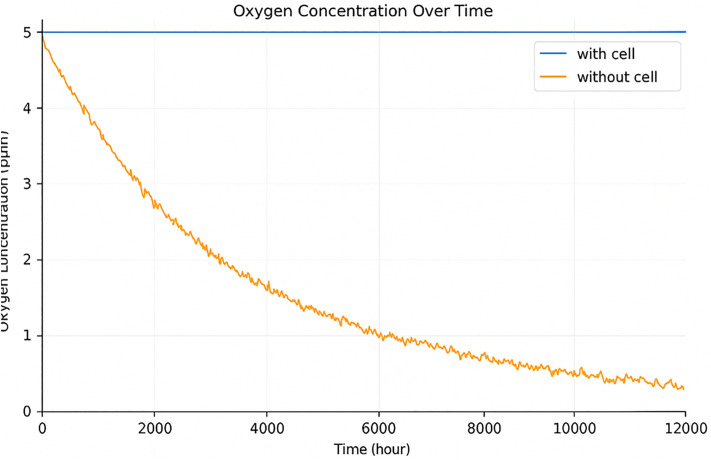
Oxygen concentration fluctuates in cancerous vs. non-cancerous regions. Pixel-wise oxygen levels plotted to demonstrate depletion caused by tumor metabolic activity and spatial constraints.

The spatial distribution of oxygen within the simulated tissue is illustrated in Figs 7 and 8.

The diffusion model generates realistic oxygen gradients, with higher concentrations near the microchannels and pronounced hypoxia in dense tumor regions. As the tumor expands, oxygen availability decreases in peripheral and overcrowded areas, leading to localized hypoxic zones that limit proliferation and promote treatment resistance. These results are consistent with experimental observations reported by Grimes et al. (2016) and Norton et al. (2014), confirming that the implemented diffusion framework successfully reproduces biologically plausible oxygen dynamics.

As shown in the diagram, oxygen concentration rises as we get closer to the center of the tissue, where tumor growth is more active. This happens because this area is nearer to the oxygen-carrying microchannels. You can also see several local peaks of higher oxygen levels right around these microchannels, which emphasizes how crucial they are for delivering oxygen throughout the simulated tissue.

In regions where cancer cells are present, oxygen levels tend to fluctuate quite a bit. When cancer stem cells are rapidly dividing and moving around, this subject is more noticeable firstly. The main reason is that fast-growing cells consume a lot of oxygen, while the oxygen supply isn’t spread out equally. This uneven demand and supply cause those ups and downs in oxygen concentration.

Such fluctuations can lead to the formation of hypoxic region within the tumor, which are known to reduce the efficacy of treatments such as radiotherapy and promote tumor resistance.

This section, in addition to analyzing oxygen behavior in various regions of the simulated tissue, we also examined the dynamic growth of cancer stem cells. Key features such as division frequency, migration rate, and resource competition were tracked, demonstrating the high adaptability of these cells to environmental constraints.

In the following section, we will present the results of applying radiotherapy to the model, evaluating its effectiveness under varying oxygenation conditions.

### Model validation using experimental data

To validate the accuracy and biological relevance of the proposed agent-based model, the simulated tumor area growth was compared with **experimental data obtained from reference [10]**:

### Experimental observation

**Fig 9:**
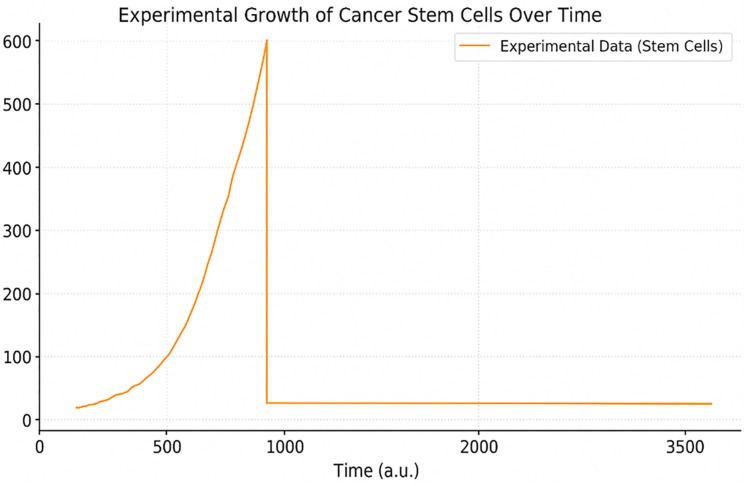
Experimental tumor growth data from Norton er al. **[10].** Shows CSC-driven tumor progression in vivo used for model validation.

At the beginning, the number of cancer stem cells stayed approximately constant that reflects a usual “quiet” period tumor development. After this phase, the cells started growing quickly and faster proliferation—almost exponentially— because they can access better nutrients and oxygen in their surroundings. The growth curve shows a terminal plateau, likely due to hypoxia, space constraints, and nutrient depletion. The experimental tumor growth data (Fig 9) show an initial slow phase followed by accelerated proliferation.

### Simulated observation

The simulated tumor area exhibited a slow initial increase then showed an accelerated growth phase. There is some Fluctuations in the simulation that reflected the stochastic behavior of individual cells, such as random proliferation, migration, and death events. First, analyzing the stability and non-divergence of simulated model shows that this model tends to stability during a time and cancer stem cells growth hasn’t overwhelmed divergence or fluctuations. Table 5 shows the analysis of numerical validation of stability and non-divergence of our proposed agent-based model.

**Table 5:** Non-divergence and stability analysis of model.

Criteria	Amount	Description
**Initial growth slope**	0.0	Almost constant
**Final growth slope**	0.0	Stable situation and growth stop
**Saturation trend**	0.0	Completely saturation of CSCs growth
**Peaks number**	1	Shows high stability of model
**Concavity number**	0	The CSC population doesn’t decrease suddenly
**Overall pattern**	stable	Model shows stable and non-divergent behavior

These results show prove that our model can be considered as a reliable frame for predicting CSCs growth in tumor environment.

Like the experimental data, the simulated growth curve flattened finally. This indicates environmental resource exhaustion, particularly oxygen deficiency. A comparison between the proposed simulation model and the **experimental setup in [10]** shown in Table 6.

**Table 6:** Key similarities and differences.

Key Similarities	Key Differences
**Growth pattern**	Temporal Scale
**Biological Drivers**	Magnitude
**Random Fluctuations**	-------------------

**Fig 10:**
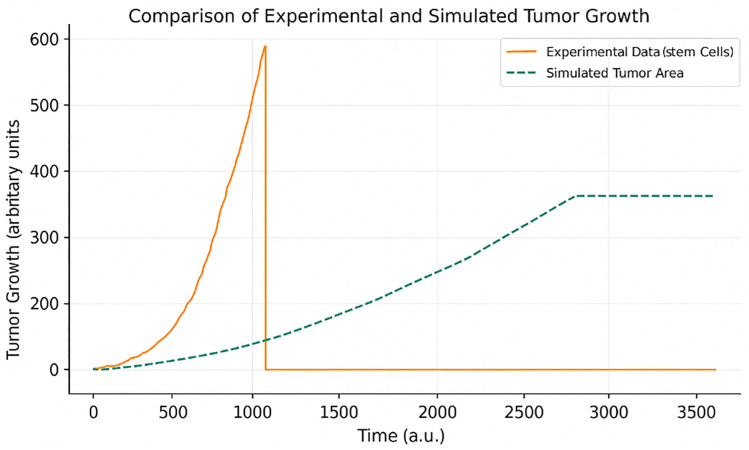
Comparison between experimental and simulated tumor area growth. Model prediction closely matches biological data in both shape and saturation behavior.

A direct comparison between the simulated and experimental tumor area is presented in Fig 10. As shown in Fig 10, the simulated tumor area closely matches the experimental curve, exhibiting similar early growth behavior and saturation dynamics:

### Numerical validation

To evaluate the accuracy and predictive power of the proposed agent-based model, the simulated cancer stem cell (CSC) population dynamics were quantitatively compared against **experimental data reported by Norton et al.** Three standard validation metrics were employed: Root Mean Square Error (RMSE), Mean Absolute Error (MAE), and the Coefficient of Determination (R^2^). RMSE measures the average magnitude of prediction error while placing a higher penalty on larger deviations; in this study, an RMSE of 74.56 indicates moderate deviation from empirical observations. MAE, which captures the average absolute difference between predicted and observed values, was calculated to be 58.46, confirming an acceptable prediction accuracy with reduced sensitivity to outliers. Most importantly, the R^2^ value reached 0.976, indicating that 97.6% of the variance in experimental CSC growth could be explained by the simulation. This high R^2^ confirms that the proposed model effectively captures the biological growth pattern of CSCs and demonstrates strong alignment with real-world tumor development behavior. As shown in Table 7, the numerical validation metrics (RMSE, MAE, and R2) demonstrate strong agreement between the simulated and experimental CSC growth curves:

**Table 7:** Numerical validation between experimental and simulation data.

Validation criterion	Amount
**RMSE**	74.56
**MAE**	58.46
$R^{2}$	0.976

### Validation conclusion

The comparison shows a clear match between the simulation results and experimental findings. Our agent-based model explores important features of tumor growth, such as the early dormant phase, rapid proliferation, random cell behavior, and reduce growth rate. These outcomes highlight the model’s value as a useful tool for simulating cancer stem cells behavior and tumor growth process under limited environmental conditions.

### Radiotherapy (ABM + RL)

To demonstrate the adaptive capability of the proposed framework, a reinforcement learning (Q-learning) algorithm was coupled with the agent-based model to guide fractionated radiotherapy. The quantitative effect of Q-learning–guided radiotherapy on CSC and non-CSC populations is summarized in Table 8. Simulation results after 480 hours indicate substantial tumor shrinkage and decreased CSC prevalence in the targeted region, as shown in Table 8 (see also S1 Table). Implementation details—including state/action definitions, reward formulation, and the tuning procedure for the weighting factors $w_{\text{1}}$and $w_{\text{2}}$—are provided in Supporting Information (S2 File), which also reports full algorithmic settings, hyperparameters, and additional numerical results.

**Table 8:** Quantitative results of Q-learning therapy.

Cell Type	Before RT	After RT	Killed
**CSC**	98	30	68
**Non-CSC (type 1)**	140	32	108
**Non-CSC (type 2)**	193	48	145

A detailed numerical comparison between the RL-guided adaptive radiotherapy and the constant-dose (2 Gy × 8) protocol is provided in S1 Table. The results confirm that the RL-based approach achieved a greater reduction in CSC count (69%) relative to baseline (98 → 30 cells), compared with the non-adaptive constant-dose schedule (65% reduction).

## Discussion

This study developed a biologically constrained agent-based model (ABM) integrated with a proof-of-concept reinforcement learning (RL) module to simulate adaptive radiotherapy for cancer stem cell (CSC)–driven tumors. Unlike static treatment models, our framework dynamically adjusts dose and beam direction according to tumor feedback, such as CSC distribution and oxygen gradients, thereby representing an early step toward biologically informed adaptive therapy.

Compared with constant 2 Gy-per-fraction radiotherapy, the RL-guided strategy achieved greater CSC suppression and more homogeneous tumor regression (see S1 Table). As shown in Table 8, the Q-learning–guided radiotherapy substantially reduced both CSC and non-CSC populations, demonstrating the adaptive advantage of the method. This advantage arises from the agent’s ability to learn spatially adaptive dosing strategies that target hypoxic or CSC-dense regions while minimizing unnecessary exposure to healthy tissue. Although the current implementation focuses on feasibility rather than full clinical optimization, the results highlight the potential of combining machine learning with mechanistic tumor models.

The model also reproduced realistic CSC-driven behaviors, including hierarchical tumor growth, oxygen-dependent proliferation, and power-law-like migration patterns. While rare, long-range CSC migrations were found to substantially influence tumor spread, supporting the hypothesis that limited motility can drive recurrence.

Several limitations remain. The simulation is two-dimensional and excludes immune and angiogenic interactions for computational simplicity. The RL module has not yet undergone full hyperparameter tuning, and validation was performed using murine rather than patient data. Future work will extend the model to 3D geometry, integrate clinical datasets, and experimentally evaluate RL-predicted radio sensitivity through in vitro clonogenic assays.

In summary, the proposed ABM-RL framework provides a computational foundation for feedback-driven, personalized radiotherapy planning and demonstrates how biologically grounded simulations can guide the development of adaptive, AI-assisted cancer treatment strategies. Table 9 summarizes key features and distinguishes our model from previous studies in terms of biological details, therapeutic modeling and simulation structure.

**Table 9:** Comparison with previous Models.

Feature/ Study	This research	Weerasinghe et al., 2024 [23]	Stephan et al., 2024 [6]	Ravoni et al., 2025 [7]	Burrage et al., 2025 [24]
**Main Focus**	CSC + Adaptive Therapy with ABM	Intertumoral Microenvironment Interactions (TME)	Review of ABM Tools in Cancer	Hepatoblastoma Modeling with Therapy	Learning Surrogate Equations for ABM
**Therapy Modeling**	Yes, with Adaptive Q-learning	No	Not Proposed	Yes, Simulated Therapy	As Surrogate Equation, Not Direct
**Numerical Validation**	Yes, with RMSE, MAE, R² = 0.976	No	No	No	No
**Sensitivity Analysis**	Yes (a00, a01, migration)	Not in detail	No	No	Separately, not clearly
**Oxygen and Hypoxia Modeling**	Yes (PDE + Gaussian)	Yes	Not Mentioned	Not Mentioned	Yes (Macro-scale)
**CSC Migration Behavior**	Modeled, with Dispersion Analysis and Power-law	Part of Model	Only General Mention	Not Mentioned	Non-ABM Based
**Scalability to Personalized Therapy**	Yes (RL-based, Parameterized)	No	Not Precisely	No	Proposed
**Model Type**	Agent-based with Biophysical Constraints	Agent-based Network	Literature Review	Specific to Hepatoblastoma	Surrogate Equation Learning
**Microenvironment Coverage**	Yes (Oxygen, Cell Space, Resource Competition)	Yes	General Suggestion	No	Yes, Partial

As illustrated, the present model provides a more comprehensive framework by simultaneously modeling CSC behavior, cell migration, treatment response, and microenvironmental interactions, while remaining computationally tractable and extensible.

### Limitations

Despite the strengths of the proposed agent-based model, several limitations should be noted. First, the simulation was conducted in a two-dimensional space, which does not fully capture the complexity of three-dimensional tumor growth and tissue interactions. Extending the model to a 3D spatial grid is a logical next step.

Second, while the model incorporates biological constraints such as oxygen diffusion and cell cycle dynamics, it omits interactions with immune cells, angiogenesis, and detailed molecular signaling pathways. These omissions were necessary for computational tractability but limit biological realism.

Third, the reinforcement learning (Q-learning) component introduced in this study is conceptual and not yet optimized for clinical scenarios. A thorough analysis of training convergence, exploration strategies, and hyperparameter tuning will be explored in future research.

Finally, the model’s validation is based on in vivo murine data rather than human clinical datasets. While the experimental results used are biologically relevant, future work should incorporate patient-specific data to increase translational applicability. While murine data provide valuable biological relevance for preliminary validation, translating these findings to human clinical settings will require parameter calibration and verification using patient-derived datasets.

### Future works

We plan to address these limitations by extending the model into three dimensions, calibrating parameters with clinical datasets, simulating non-uniform radiation fields, and enhancing the reinforcement learning module to support real-time, patient-specific radiotherapy planning. In addition, future in vitro experiments will be designed to evaluate the radiotherapeutic sensitivity predicted by the RL-guided framework and further validate its biological applicability.

## Supporting information

S1 Table Comparative cancer stem cell (CSC) outcomes between reinforcement learning (RL)–guided adaptive radiotherapy and constant-dose (2 Gy × 8) radiotherapy. CSC values are averaged over multiple random seeds.
(DOCX)

S2 File Reinforcement learning–guided adaptive radiotherapy module. Detailed description of the Q-learning implementation, including state and action definitions, reward formulation, hyperparameters, and additional numerical results.
(DOCX)
