# Supplementary material for: A biologically constrained agent-based model of cancer stem cell dynamics with reinforcement learning-guided adaptive radiotherapy
Source: PLoS One. 2026 Feb 5;21(2):e0340426. doi: 10.1371/journal.pone.0340426 (PMC12875451; doi:10.1371/journal.pone.0340426)
Supplement: S1 Table — CSC values are averaged over multiple random seeds. (DOCX) [file pone.0340426.s001.docx]

# S1 Table. Comparative CSC outcomes between RL-guided adaptive radiotherapy and constant-dose RT:

CSC values averaged across multiple random seeds. The reinforcement learning (RL) agent adaptively selects radiation dose and beam direction based on the spatial distribution of cancer stem cells (CSCs) and oxygen feedback. Results demonstrate consistently lower surviving CSCs in the RL-guided protocol compared to the fixed 2 Gy-per-fraction treatment.

**Table 1- comparative CSC outcomes between RL-guided adaptive radiotherapy and constant-dose RT**

| Method | CSC (mean ± SD) | % reduction vs start |
| --- | --- | --- |
| RL (Adaptive) | 30 ± 2.1 | 69% |
| Constant-dose RT (8×2 Gy) | 34 ± 2.4 | 65% |

This supplementary table supports the results section of the main manuscript, providing quantitative evidence of the adaptive advantage of RL-guided radiotherapy in reducing cancer stem cell (CSC) survival compared to a constant-dose protocol.
