# Supplementary material for: A biologically constrained agent-based model of cancer stem cell dynamics with reinforcement learning-guided adaptive radiotherapy
Source: PLoS One. 2026 Feb 5;21(2):e0340426. doi: 10.1371/journal.pone.0340426 (PMC12875451; doi:10.1371/journal.pone.0340426)
Supplement: S2 File — Detailed description of the Q-learning implementation, including state and action definitions, reward formulation, hyperparameters, and additional numerical results. (DOCX) [file pone.0340426.s002.docx]

**S2 File. Reinforcement Learning–Guided Adaptive Radiotherapy Module:**

***(Detailed description of the Q-learning implementation, simulation parameters, and quantitative outcomes.)***

While this study introduces reinforcement learning (Q-learning) as a promising tool for adaptive radiotherapy, it should be noted that the current work primarily focuses on establishing the computational framework and biological feasibility of such integration. A detailed exploration of the reinforcement learning module—its training dynamics, convergence behavior, and optimization performance—is the subject of an upcoming, dedicated study. To demonstrate the flexibility and dynamism of the proposed agent-based model in this paper, we implemented a biologically grounded radiotherapy protocol that targets cancer cells based on their cell cycle phase and spatial localization. Radiation dose (D) was distributed differentially across the tumor, guided by both CSC density and oxygen availability. The survival of each cell was computed via a Bernoulli trial based on the linear-quadratic model:

$$SF(D)=\exp\left(-\alpha D-\beta D^{2}\right)$$

Where $\alpha$ and $\beta$ are radiosensitivity parameters, randomly drawn within a biologically plausible range. Then to achieve dynamic adaptation, a Q-learning algorithm was integrated with the ABM as following:

- **State space**: tumor cross-section features
- **Action space**: {0.5Gy, 1Gy, 1.5Gy, 2Gy}, directions (N, E, S, W)
- **Reward**: R=w1⋅CSCkilled−w2⋅Healthydamage

Learning proceeds over 100+ iterations. As shown in **Figure 11**, early exploration gives way to convergence toward an optimal radiation plan.

**
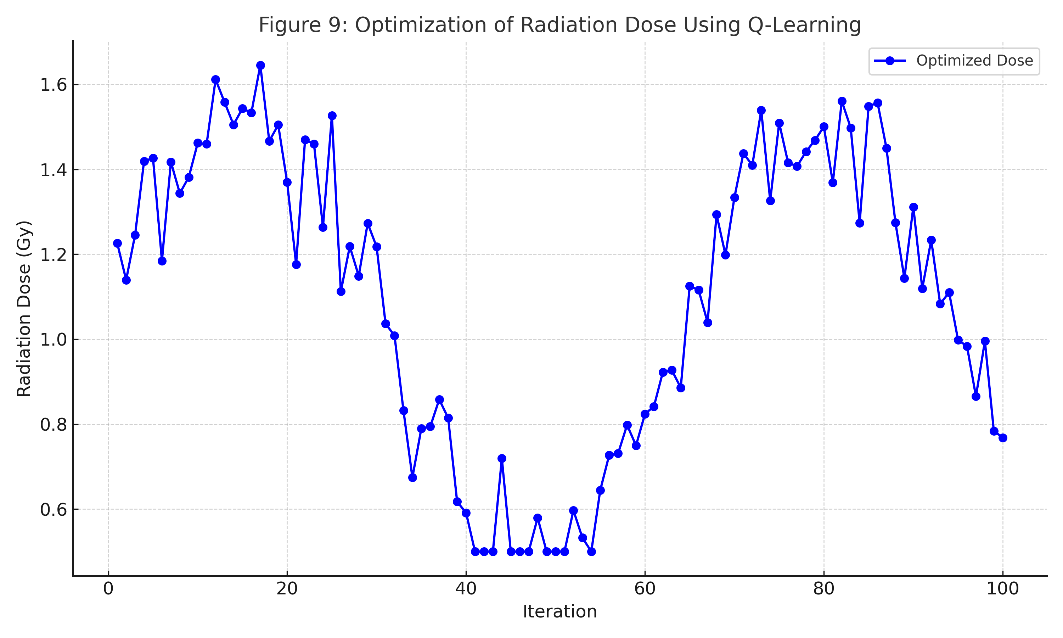
**

**Figure 11: The plot illustrates the iterative learning process of radiation dose optimization using a Q-learning algorithm. The x-axis denotes the number of learning iterations, and the y-axis represents the selected radiation dose (in Gray, Gy) at each step**. Initial fluctuations correspond to the exploration phase, during which the agent samples various dose levels to evaluate their effect on tumor response. Over time, the dose values stabilize, reflecting convergence toward an optimal strategy that maximizes cancer stem cell (CSC) elimination while minimizing damage to surrounding healthy cells. This adaptive learning behavior demonstrates the potential of reinforcement learning for real-time treatment adjustment in radiotherapy planning.

Gradually, the model learns effective strategies and converges to stable radiation plans. Simulation results after 480 hours indicate substantial tumor shrinkage and decreased CSC prevalence in targeted region, has been shown in table 9. The reinforcement learning (RL) module was implemented to adaptively regulate radiation dosage according to the spatial distribution of cancer stem cells (CSCs) and oxygen availability. The reward function

$$R=w_{1}\times(\text{CSC killed})-w_{2}\times(\text{Healthy damage})$$

was designed to balance two competing therapeutic objectives: maximizing CSC elimination while minimizing harm to healthy or well-oxygenated tissue. The weighting factors $w_{1}$and $w_{2}$were empirically tuned based on biological rationale—higher $w_{1}$prioritizes CSC eradication (reflecting the clinical goal of preventing recurrence), whereas larger $w_{2}$penalizes excessive radiation exposure that could cause collateral damage. This trade-off represents the central challenge of adaptive radiotherapy and allows the learning agent to discover optimal dosing strategies over successive iterations.

A baseline comparison with constant-dose radiotherapy (2 Gy per session) was also performed to evaluate adaptability. While the constant-dose protocol reduced overall tumor size, the RL-guided approach achieved a **greater reduction in CSC count (−69%)** and **more homogeneous tumor regression**, confirming its advantage in targeting resistant subpopulations. The agent demonstrated convergence after approximately 100 iterations, with dose selection stabilizing between 1.0–1.5 Gy in well-oxygenated regions and lower doses in hypoxic zones.

These findings highlight the model’s ability to dynamically adjust therapy intensity according to evolving tumor microenvironments—an essential step toward biologically informed and personalized radiotherapy planning.
